# An Investigation into the Genetic History of Japanese Populations of Three Starfish, *Acanthaster planci*, *Linckia laevigata*, and *Asterias amurensis*, Based on Complete Mitochondrial DNA Sequences

**DOI:** 10.1534/g3.120.401155

**Published:** 2020-05-29

**Authors:** Jun Inoue, Kanako Hisata, Nina Yasuda, Noriyuki Satoh

**Affiliations:** * Marine Genomics Unit, Okinawa Institute of Science and Technology Graduate University, Onna, Okinawa 904-0495, Japan and; ^†^ Marine Molecular Ecology Laboratory, Faculty of Agriculture, University of Miyazaki, Miyazaki 889-2192, Japan

**Keywords:** Three starfish, genetic history of Japanese populations, complete mitochondrial DNA sequence, characteristic profile of COTS population genetics, repeated bottleneck phenomena

## Abstract

Crown-of-thorns starfish, *Acanthaster planci* (COTS), are common in coral reefs of Indo-Pacific Ocean. Since they are highly fecund predators of corals, periodic outbreaks of COTS cause substantial loss of healthy coral reefs. Using complete mitochondrial DNA sequences, we here examined how COTS outbreaks in the Ryukyu Archipelago, Japan are reflected by the profile of their population genetics. Population genetics of the blue starfish, *Linckia laevigata*, which lives in the Ryukyu Archipelago, but not break out and the northern Pacific sea star, *Asterias amurensis*, which lives in colder seawater around the main Islands of Japan, were also examined as controls. Our results showed that *As. amurensis* has at least two local populations that diverged approximately 4.7 million years ago (MYA), and no genetic exchanges have occurred between the populations since then. *Linckia laevigata* shows two major populations in the Ryukyu Archipelago that likely diverged ∼6.8 MYA. The two populations, each comprised of individuals collected from coast of the Okinawa Island and those from the Ishigaki Island, suggest the presence of two cryptic species in the Ryukyu Archipelago. On the other hand, population genetics of COTS showed a profile quite different from those of *Asterias* and *Linckia*. At least five lineages of COTS have arisen since their divergence ∼0.7 MYA, and each of the lineages is present at the Okinawa Island, Miyako Island, and Ishigaki Island. These results suggest that COTS have experienced repeated genetic bottlenecks that may be associated with or caused by repeated outbreaks.

We are interested in genic, genetic, and genomic changes that underly outbreaks of crown-of-thorns starfish (COTS), *Acanthaster planci*, especially in the Ryukyu Archipelago, southwestern and subtropical islands of Japan. COTS are common in coral reefs throughout the Indo-Pacific Ocean and are highly fecund predators of reef-building corals (Birkeland and Lucas 1990; Fabricius 2013). COTS periodically break out, which causes substantial loss of coral cover, diminishing the integrity and resilience of reef ecosystems (De’ath *et al.* 2012; Pratchett *et al.* 2014; Uthicke *et al.* 2015). In the Great Barrier Reef (GBR), Australia, one-third of coral-reef damage is attributed to COTS predation (De’ath *et al.* 2012). The Ryukyu Archipelago comprises of three main islands, from south to north: Ishigaki Island, Miyako Island, and Okinawa Island. Although the strong Kuroshio Current runs northward along the archipelago, local surface currents in Okinawa Prefecture appear more complex (the Japan Meteorological Agency: http://www.jma.go.jp/jma/indexe.html), presumably affected by winds and other factors. In the Ryukyu Archipelago, the first COTS outbreak was recorded in the late 1950s near Ishigaki Island and then expanded northeastward to Okinawa Island, followed by large, periodic outbreaks (Yamaguchi 1986; Nakamura *et al.* 2014). For example, during the 1980s, more than 1.5 million COTS were removed by divers, in a very painful and costly effort to maintain healthy coral reefs. Although COTS are presently decreasing in number in the Ryukyu Archipelago, severe local outbreaks have been reported in recent years, and others are anticipated in the future (Dr. Ken Okaji, personal communication).

Why and how do such periodic outbreaks of COTS occur? Adult COTS are large, reaching ∼30 cm in diameter, and they spawn hundreds of thousands of comparatively small eggs. It is thought that anthropogenic environmental changes, such as pollution, increased temperatures, and especially eutrophication of seawater, increase survival of COTS larvae and juveniles, promoting outbreaks (Pratchett *et al.* 2017). However, many questions remain unanswered regarding reproductive, developmental, and ecological mechanisms of COTS outbreaks. To address these questions, several population genetic studies have been performed in various regions of Indo-Pacific Ocean (Nishida and Lucas 1988; Yasuda *et al.* 2009). For example, Vogler *et al.* (2012) identified the geographic distribution of two COTS lineages in the Indian Ocean based on partial mitochondrial gene sequences. By using mitochondrial DNA and microsatellite loci, Yasuda *et al.* (2015) reported genetically homogenized pattern of COTS during an outbreak from 2006-2009 in French Polynesia. Between ‘reef-scale’ populations in the central Pacific Ocean, although Timmers *et al.* (2012) observed genetic differentiation of mitochondrial control region, they also found vestiges of recent gene flow or the ancestral polymorphisms/gene flow. In GBR, a microsatellite survey by Harrison *et al.* (2017) suggested high genetic homogeneity in populations from four different locations. These researches suggest limited dispersal of COTS within smaller geographic areas. On the other hand, using microsatellites, Tusso *et al.* (2016) documented the lack of genetic differentiation between Guam and extremely distant populations in the Pacific Ocean. The potential long-distance dispersal of COTS in the Pacific Ocean indicates that COTS outbreaks can be caused by recent colonization from different Pacific populations.

Our previous study sequenced the genomes of two wild-caught COTS from locations separated by over 5,000 km, one from the GBR and the other from the Ryukyu Archipelago (Hall *et al.* 2017). The ∼384-Mb draft assembly was estimated to contain ∼25,400 protein-coding genes. Interestingly, heterozygosity of the genomes was unexpectedly low, 0.88% and 0.92% for the GBR and the Ryukyu Archipelago, respectively. In addition, reciprocal BLAST analysis of scaffolds longer than 10 kb showed 98.8% nucleotide identity between the GBR and the Ryukyu Archipelago genomes, evidence of the great similarity of their nuclear DNA sequences. This great nuclear DNA sequence similarity in marine invertebrates from such widely separated locations is extremely unusual.

In this study, we examined population genomics of COTS in the Ryukyu Archipelago from a different perspective than those of previous studies. First, we employed a technical improvement. In general, population genetic studies of marine invertebrates have been carried out by comparing sequences derived from microsatellites or sequences of a few genes, including the mitochondrial cytochrome C oxidase subunit 1 gene (*COI*). Given the sequence similarity of the two COTS mentioned above, sequence comparisons of single genes or a limited number thereof, might not yield useful results. Therefore, the present study analyzed complete mitochondrial DNA sequences. Second, we examined two other species, which were used as controls. For species boundaries of marine invertebrates, molecular data have revealed enigmatic instances (Avise 2000): (1) a widespread, phenotypically variable “species” is actually several sympatric or parapatric populations of sibling taxa, or (2) taxonomically accepted species are genetically indistinguishable from each other. Analyses using only COTS can offer limited information about the evolutionary history that underlies recent outbreaks. So, we thought employment of other sea star species for this purpose. The class Asteroidea is one of the most diverse groups in the phylum Echinodermata, including nearly 1,900 extant species (Mah and Blake 2012). We also examined the blue starfish, *Linckia laevigata*, which inhabits the same coral reefs as COTS in the Ryukyu Archipelago, but *L. laevigata* never exhibits break out, and the northern Pacific sea star, *Asterias amurensis*, which is common in the colder waters of the Japanese main islands, occurring in environmental conditions very different from coral reefs.

## Materials And Methods

### Biological materials

Three species of starfish, *Asterias amurensis*, *Linckia laevigata*, and *Acanthaster planci* were examined in this study. Collection localities and sample numbers are summarized in Table 1.

**Table 1:** Three starfish used for this population genomics study

Species name	Location	Number	Approximate latitude	Year
*Asterias amurensis*	Ushimado	10	34°60’ N, 134°14’ E	2017
	Onagawa	5	38°44’ N, 141°46’ E	2018
	Asamushi	10	40°89’ N, 140°86’ E	2017
*Linckia laevigata*	Okinawa	10	26°49’ N, 127°83’ E	2018
	Ishigaki	15	24°20’ N, 124°10’ E	2018
*Acanthaster planci*	Okinawa	12	26°49’ N, 127°83’ E	2017
	Miyako	6	24°84’ N, 125°31’ E	2017
	Iriomote	8	24°33’ N, 123°73’ E	2017

### Asterias amurensis

Twenty-five adult *Asterias amurensis* were collected from three locations of the main island of Japan (Table 1); 10 specimens from Mutsu Bay, near the Asamushi Research Center for Marine Biology of Tohoku University, Aomori in fall of 2017, five from Onagawa Bay near Onagawa Field Center of Tohoku University, Miyagi in winter of 2018, and 10 from the Seto Inland Sea, near the Ushimado Marine Institute of Okayama University, Okayama in winter of 2017.

### Linckia laevigata

Twenty-five adult *Linckia laevigata* were collected in the summer of 2018 in the South China Sea, in the Ryukyu Archipelago (Table 1). These included 10 from Onna, Okinawa Island and 15 from Ishigaki Island.

### Acanthaster planci

Twenty-six adult *Acanthaster planci* were collected in the summer of 2017 in the South China Sea, from three locations (Table 1). Twelve were collected at Onna, Okinawa Island, six from Miyako Island, and eight from Iriomote Island.

### DNA sequencing and assembly of mitochondrial genomes

Feet of adults were dissected with scissors and fixed in 80% ethanol. Specimens were kept at 4° until use for DNA sequencing. Genomic DNA was extracted from a total of 76 specimens using the standard phenol-chloroform method with 100 mg/L RNaseA treatment. The quantity of DNA was determined by NanoDrop (Thermo Scientific Inc., Madison, USA), and the quality of high molecular-weight DNA was checked using agarose gel electrophoresis.

In paired-end library preparations for sequencing, genomic DNA was fragmented with Focused-ultrasonicator M220 (Covaris Inc., Massachusetts, USA). Paired-end libraries (average insert size: 540 bp) were prepared using Illumina TruSeq DNA LT Sample Prep Kits (Illumina Inc., San Diego, USA), following the manufacturer’s protocols. Sequencing was performed using the Illumina HiSeq 2500 sequencer. Approximately 30X coverage of nuclear genome DNA sequences were obtained. After removing low-quality reads, under default parameters, paired-end reads were assembled using GS *De novo* Assembler version 2.3 (Newbler, Roche) and NOVOPlasty 2.6.3 (Dierckxsens *et al.* 2016) with published *A. planci* sequence (Hall *et al.* 2017) as the seed input. Usually, the largest scaffolds contained mitochondrial DNA sequences.

### Alignment

Whole mitochondrial genome sequences were aligned using MAFFT (Katoh *et al.* 2005). Multiple sequence alignments were trimmed by removing poorly aligned regions using TRIMAL 1.2 (Capella-Gutierrez *et al.* 2009) with the option “gappyout.”

### Divergence time estimation

To determine the approximate timing of divergence events, a time calibrated tree was estimated using RelTime (Tamura *et al.* 2012), as implemented in MEGA X (Kumar *et al.* 2018). To estimate divergence times, a neighbor-joining tree (Saitou and Nei 1987) was estimated using a dataset comprising all 76 specimens of the three species, using the GTR model with gamma-distributed rate variations among sites (Yang 1994a; Yang 1994b). In order to bisect possible long branches, data of sister species were added for focal species by using retrieved sequences from NCBI: *Aphelasterias japonica* for *Asterias amurensis*, *Luidia quinaria* and *Patiria pectinifera* for *Linckia laevigata*, and *Acanthaster brevispinus* for *Ac. planci* (Table 2). *Ophiacantha linea* and *Gorgonocephalus chilensis* (Ophiuroidea) were used for rooting (Table 2). The previously estimated divergence time between *Luidia* (Luidiidae) and *Patiria* (Asterinidae), ca. 185 MYA (O’Hara *et al.* 2014), was employed as a calibration point. All positions containing gaps and missing data were eliminated. Mitochondrial DNA sequence data used for analyses are summarized in Table 2.

**Table 2:** Classification and data source of species analyzed

Classification	Data source
Asterozoa	
Ophiuroidea	
Ophiuridea	
Euryalida	
Gorgonocephalidae	
* Gorgonocephalus chilensis*	NC_040147.1
(Basket star)	
Ophiurida	
Ophiacanthidae	
*Ophiacantha linea*	KC990833.1
(Brittle star)	
Asteroidea	
Forcipulatacea	
Forcipulatida	
Asteriidae	
* Aphelasterias japonica*	NC_025766.1
* Asterias amurensis*	This study
(Northern Pacific sea star)	
Valvatacea	
Paxillosida	
Luidiidae	
*Luidia quinaria*	NC_006664.1
(Spiny sand sea star)	
Valvatida	
Asterinidae	
* Patiria pectinifera*	NC_001627.1
(Blue bat star)	
Ophidiasteridae	
* Linckia laevigata*	This study
(Blue starfish)	
Acanthasteridae	
* Acanthaster brevispinus*	NC_007789.1
(Short-spined crown-of-thorns starfish)	
* Acanthaster planci*	This study
(Crown-of-thorns starfish)	

### Phylogenetic analysis

To examine population structures within each species, maximum likelihood (ML) trees were created using RAxML 8.2.6 (Stamatakis 2014). Trees were estimated with the “-f a” option, which invokes a rapid bootstrap analysis with 100 replicates and searches for the best-scoring ML tree, using the GTRCAT model.

### Principal component analysis (PCA)

We also analyzed population structures using model-free approaches. Based on mitochondrial genome sequences, principal component analysis (PCA) was performed on all individuals, using PLINK 1.9 (Chang *et al.* 2015). Pairwise genetic distances among localities were estimated with Weir and Cockerham’s *F*_ST_ (Weir and Cockerham 1984) and Nei’s genetic distance (Nei 1972) using StAMPP (Pembleton *et al.* 2013).

### Data availability

Figure S1 contains alignment of the complete mitochondrial DNA sequences of *Acanthaster planci* (NC_007788.1 and specimen’s name, M2), *Linckia laevigata* (specimen’s name, Ishigaki-11), and *Asterias amurensis* (specimen’s name, A1). All the sequence data are accessible under https://www.ncbi.nlm.nih.gov/bioproject/PRJDB9380. Supplemental material available at figshare: https://doi.org/10.25387/g3.12386060.

## Results And Discussion

### Mitochondrial genome sequences of starfish

**Figure 1:**
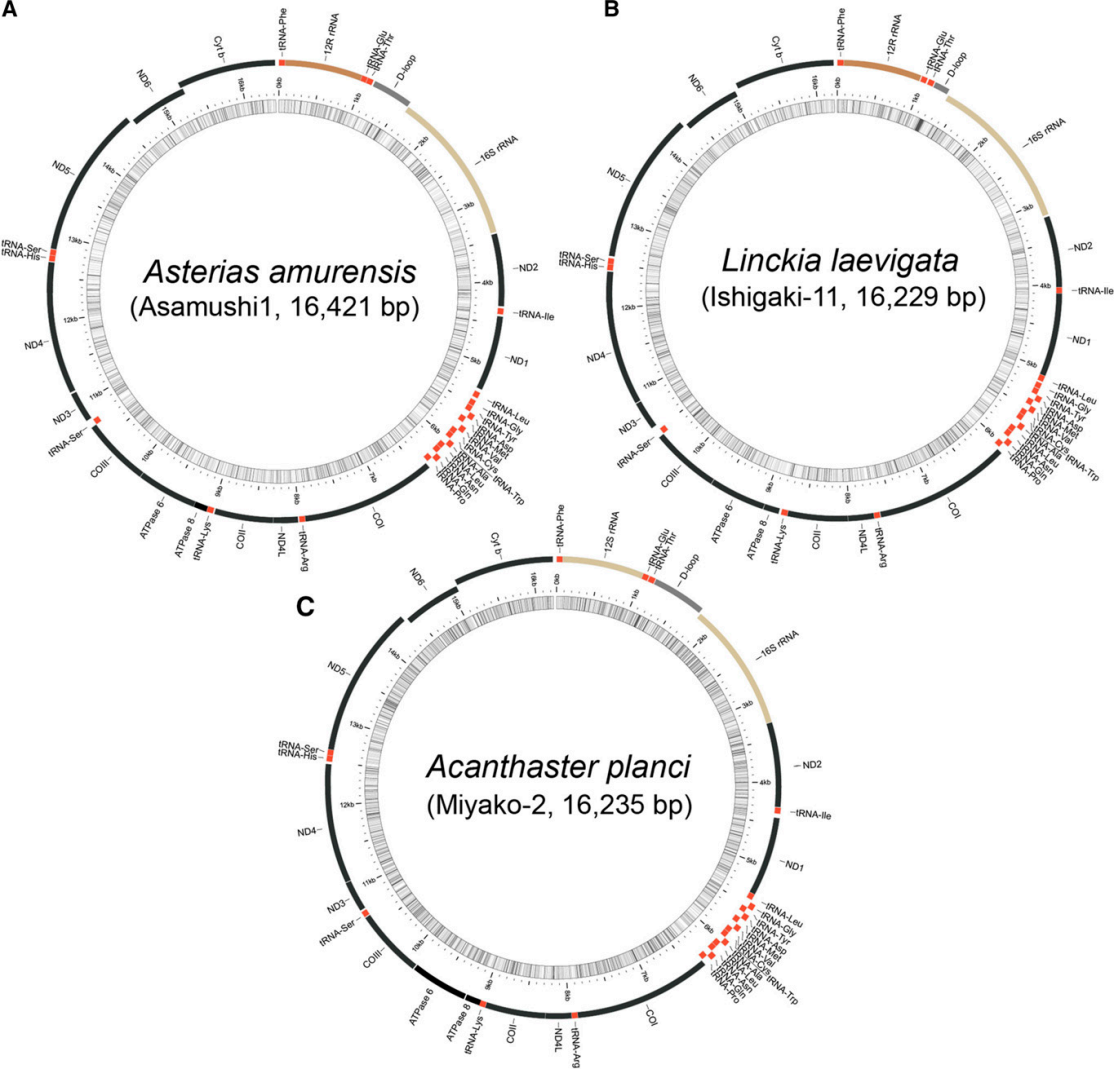
Complete mitochondrial genomes for (a) *Asterias amurensis*, (b) *Linckia laevigata*, and (c) *Acanthaster planci*, determined by whole-genome shotgun sequencing. The mitochondrial genome of *As. amurensis* is identical to that determined using a primer-based, long PCR method (Matsubara *et al.* 2005). The *L. laevigata* mitochondrial genome was sequenced in this study. The *Ac. planci* genome, which was determined using a primer-based, long PCR method (Yasuda *et al.* 2010), was improved by whole-genome shotgun sequencing in this study. Genome lengths and the order of genes are shown. Names of sequenced specimens are shown. Schematic genome structures were drawn by using MitoAnnotator (http://mitofish.aori.u-tokyo.ac.jp/annotation/input.html).

Matsubara *et al.* (2005) determined the complete mitochondria DNA sequence of an individual *Asterias amurensis* collected from the coast near Miyako City, Iwate using a primer-based long PCR method (accession #AB183559.1). It was a circular genome composed of 16,427 bp. In this study, we re-sequenced it using the whole-genome shot-gun method, with a specimen collected from Asamushi (Figure 1a). The sequences fall within the range of 16,419 to 16,421 bp and are identical to that reported by Matsubara *et al.* (2005) with respect to gene order and transcription direction. That is, the genome consists of a gene set of cytochrome oxidase subunits I, II and III (*COI*, *COII* and *COIII*), cytochrome b (*Cyt b*), NADH dehydrogenase subunits 1-6 and 4L (*ND1-6* and *4L*), ATPase subunits 6 and 8 (*ATPase6* and *8*), two rRNAs, and 22 tRNAs (Figure 1a; the entire DNA sequence is shown in Supplementary Fig. S1). The complete mitochondrial DNA sequence of *Linckia laevigata* was first determined in this study, using a specimen collected from Ishigaki (Figure 1b). Genomes of 16,211–16,365 bp contain a set of genes with identical gene order and transcription direction as in *As. amurensis* (Figure 1b; Fig. S1).

The complete mitochondrial DNA sequence of *Acanthaster planci* determined by the long PCR method was reported by Yasuda *et al.* (2006) (accession #NC_007788.1). However, the sequence we determined here, using the whole-genome shotgun method, for a specimen collected from Miyako, was not identical to that of Yasuda *et al.* (2006) (Figure 1c; Fig. S1). Even in 1^st^ or 2^nd^ codon positions, nucleotide differences were observed between the two studies (see Fig. S1). We first thought that these discrepancies might be due to individual differences, but comprehensive comparisons of the previous and present *A. planci* mitochondrial DNA sequences with those of *Asterias amurensis*, and *Linckia laevigata* suggested some reading errors in the previous study, probably due to long PCR amplification (see Fig. S1). We therefore used the present sequences (Figure 1c) for further analyses.

### Estimated divergence times of three starfish

**Figure 2:**
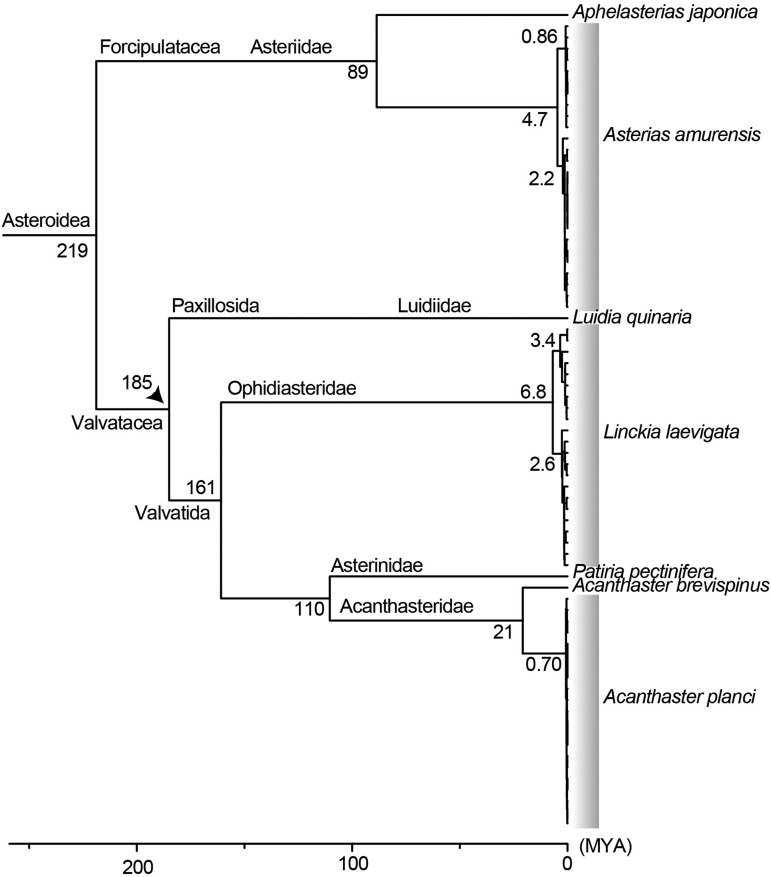
Divergence time estimation of sea star populations based on the RelTime method. An arrowhead indicates the separation between *Luidia* (Luidiidae) and *Patiria* (Asterinidae) used as the calibration point. According to O’Hara *et al.* (2014), the Paxillosida (*Luidia*) and Valvatida (*Linckia* and *Acanthaster*) separated around 185 MYA. In our time-calibrated tree, *Linckia* shows the deepest divergence. *Asterias* was more recent, and *Acanthaster* represents the newest lineage. The long branch following the diversification of *A. planci* and *A. brevispinus* (21 MYA) suggests repeated genetic bottlenecks in *A. planci*.

To compare evolutionary histories among the three starfish, we estimated divergence times of nodes, with special reference to their basal separations. Data sources for this analysis are shown in Table 2. The genus *Luidia* belongs to the order Paxillosida and the genera *Linckia* and *Acanthaster* to the order Valvatida. O’Hara *et al.* (2014) estimated that the divergence of the two orders occurred approximately 185 million years ago (MYA). Using this divergence date and by comparing complete mitochondrial DNA sequences, we estimated the divergence times of the three starfish. First, within the order Forcipulatida, comparison of the mitochondrial genome sequences of *Asterias amurensis* and *Aphelasterias japonica* suggested that these taxa diverged approximately 89 MYA. From the ancestor of *As. amurensis*, the population we examined in this study diverged 4.7 MYA, then one lineage diverged 2.2 MYA and the other 0.86 MYA (Figure 2; see following sections).

The Superorder Valvatacea is divided into the Orders Paxillosida and Valvatida, the latter of which includes *Linckia* (Family Ophidiasteridae) and *Acanthaster* (Family Acanthasteridae). Analyses of data, including those of *Luidia quinaria*, *Patiria pectinifera* and *Acanthaster brevispinus*, showed that the divergence time of a common ancestor of the two populations of *Linckia laevigata* was estimated to be 6.8 MYA. Since then, one population diverged 3.4 MYA and the other 2.6 MYA (Figure 2; see following sections). In contrast, the divergence date of *Acanthaster planci* ancestor of the populations was more recent, 0.7 MYA (Figure 2; see following sections).

### Population genetics of the three starfish

#### Asterias amurensis:

**Figure 3:**
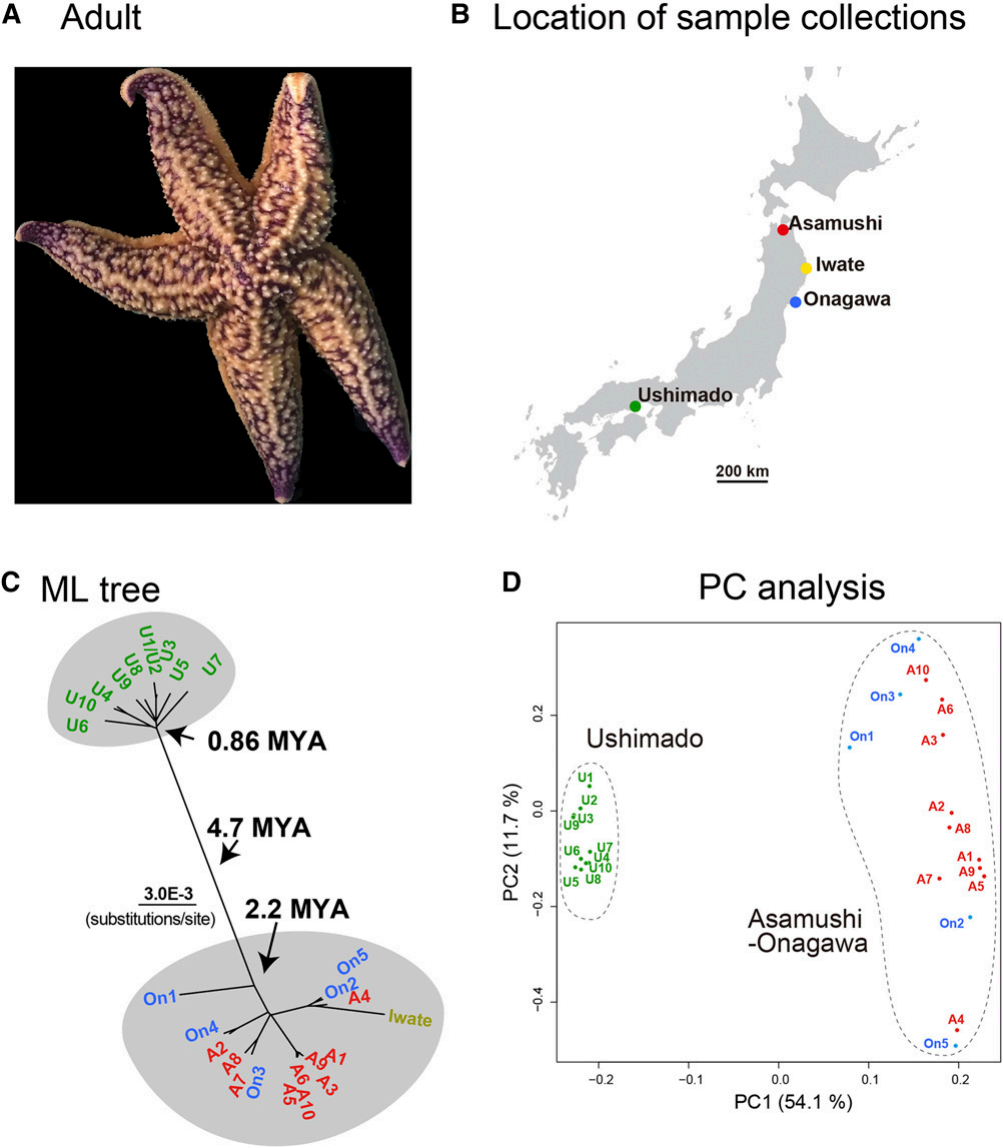
Population genetics of *Asterias amurensis*. (a) An adult. (b) Location of sample collections in the main Islands of Japan. (c) Population genetics showing two different populations that diverged approximately 4.7 MYA. Genetic flow is seen in the Tohoku population while no genetic flow is apparent in the Ushimado population. (d) PCA of the populations, supporting the population genetic profile shown in (c).

*Asterias amurensis* is common in cold-water off the coast of Japan (Figure 3a). We collected 10, 5, and 10 specimens near Asamushi, Onagawa, and Ushimado, respectively (Figure 3b), and determined their complete mitochondrial DNA sequences. The entire mitochondrial genome of *As. amurensis* was 16,419–16,421 bp in length. After removing a poorly aligned 42-bp region from a 16,458-bp alignment, we compared sequences of 16,416 nucleotides from 26 individuals, including that reported by Matsubara *et al.* (2005). Among them, 728 nucleotide sites were identified as variable sites and were able to use for ML tree analysis. Two individuals, U1 and U2, collected at Ushimado showed completely identical sequences, and therefore U2 was included in further phylogenetic analysis (Figure 3c).

This study showed the divergence of two local populations, one is from Ushimado (U: Seto Inland Sea) and the other from Tohoku (northeastern Japan) (A, Asamushi and On, Onagawa) including the specimen from Miyako, Iwate of Matsubara *et al.* (2005). The ancestor of the two populations was estimated to have diverged approximately 4.7 MYA (Figure 2; Figure 3c). The Tohoku population was established 2.2 MYA, while the Ushimado population became independent more recently, 0.86 MYA. Nine individuals from Ushimado formed a comparatively cohesive group, suggesting no genetic introgression from other areas, partially because of the isolation of the Seto Inland Sea. It is likely that the *As. amurensis* ancestor, which lived in the cooler seas of northern Japan, expanded its niche into the Seto Inland Sea around 0.9 MYA and then remained there. Since then, the Ushimado population has become independent, without genetic influx from other areas. PCA supported results of the tree analysis, namely the existence of a distinctive Ushimado population and a genetically more diverse Tohoku population (Figure 3d).

On the other hand, the Tohoku population appeared as a more diverse genetic assemblage from Asamushi, Miyako, and Onagawa. Six individuals of Asamushi formed a group, suggesting the presence of a population of *As. amurensis* restricted to Mutsu Bay. On the other hand, several groups comprised individuals from Asamushi and Onagawa, and Miyako as well (Figure 3c), suggesting frequent gene flow among these areas. A short branch including individuals from different coastal regions, *e.g.*, A2 and On4 or A8/A7/On3 suggests recent genetic exchange between Asamushi and Onagawa. Ocean currents in Tohoku are complex (Unoki and Kubota 1996), including a strong flow from Onagawa northward, a flow from Hokkaido southward, and a flow from the Japan Sea eastward through Mutsu Bay to the Pacific Ocean. Such complex sea flows might result in distinct populations in different localities.

Overall, *Asterias amurensis* population genomics appears similar to those reported for other marine animals (Hirase and Ikeda 2014). It is highly likely that after divergence of the Ushimado and Tohoku populations, the two developed unique genomic structure in the absence of gene flow between them.

#### Linckia laevigata:

**Figure 4:**
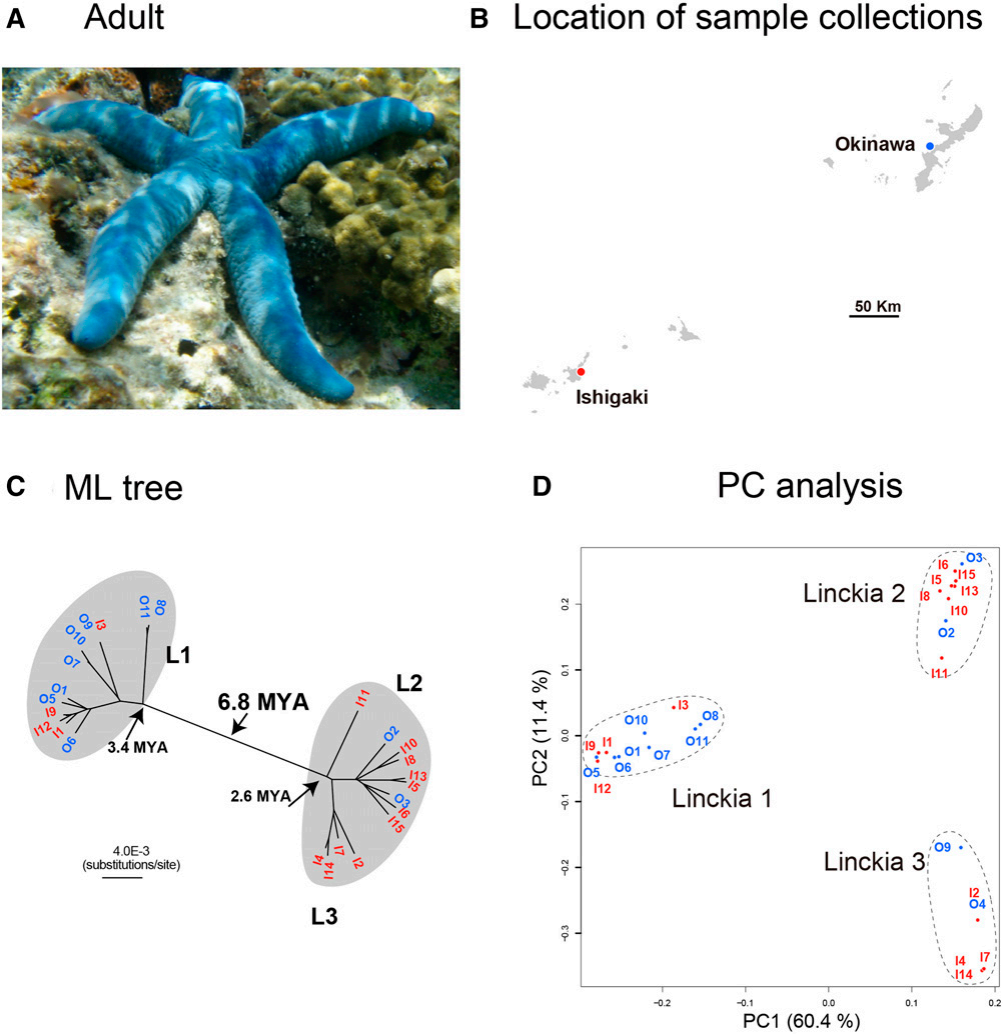
Population genetics of *Linckia laevigata*. (a) An adult. (b) Location of sample collections in the Ryukyu Archipelago, Japan. (c) Population genetics showing two different populations that diverged approximately 6.8 MYA. The two populations each comprise mixtures of individuals from Okinawa and Ishigaki Islands. (d) PCA of the populations, supporting the population genetic profile shown in (c).

*Linckia laevigata* (Figure 4a), the blue starfish, is common in the Ryukyu Archipelago, comprising from south to north, Ishigaki Island, Miyako Island, and Okinawa Island (Figure 4b). Ishigaki is approximately 400 km southwest of Okinawa, and the powerful Kuroshio Current moves northward along the three Islands. As previously mentioned, *L. laevigata* shares its coral reef habitat with COTS. *Linckia laevigata* also eats corals, but there have been no records of any outbreaks in this area in the last 70 years.

We determined the complete mitochondrial DNA sequences (16,171–17,055 bp) of 10 and 15 specimens collected form Okinawa and Ishigaki Islands, respectively. With the alignment including 1,392 variable sites, we analyzed population genomics of *L. laevigata*. The results showed that these 25 individuals were divided into two major groups, L1 and L2/L3, the former probably diverged ∼3.4 MYA and the latter 2.6 MYA (Figure 4c). L2/L3 is one of the two major groups, composed of two smaller subgroups, L2 and L3. PCA supported the results of the tree analysis with regard to populations L1 and L2/L3 (Figure 4d).

Interestingly, both populations constituted mixtures of individuals collected from Ishigaki and Okinawa. L1 was represented by eight Okinawan (O) individuals and four Ishigaki (I) individuals. L2 included two specimens from Okinawa and six from Ishigaki, while L3 consisted of four Ishigaki (Figure 4c). This result raises several issues. First, 6.8 MY have elapsed since the two populations comprising individuals from different islands (L1 and L2/L3) diverged. In other words, gene flow or genetic exchange appears not to have occurred to an appreciable degree for more than 6 MY. Crawford and Crawford (2007) reported the presence of two cryptic species of another *Linckia* species, *L. multifora*, in the Cook Islands, and Williams (2000) suggested the presence of possible cryptic species within *L. laevigata* specimens obtained from widely separated locations of the Pacific Ocean. Our results strongly suggest the presence of two cryptic populations of *L. laevigata* in the Ryukyu Archipelago, although no adult morphological differences have been reported to date.

Second, population genomic structure of *L. laevigata* may provide further insight into the role of surface currents in establishment of marine invertebrate populations. Significant gene flow among individuals from Ishigaki and Okinawa is evident in both the L1 and L2 populations. In addition, a comparatively short branch length between O5 and I9 suggests recent genomic exchange within the L1 population. It is generally believed that the northbound Kuroshio Current strongly controls the movement of marine invertebrate larvae from Ishigaki to Okinawa. If this is the case, population genomics studies may eventually reveal a pattern of two populations, one composed only of individuals from Ishigaki and the other of specimens from both Ishigaki and Okinawa. L2/L3 likely illustrates this situation, in which L3 is composed of only Ishigaki individuals while L2 is a mixture of both islands in the ML tree analysis.

On the other hand, L1 is composed of a mixture of Ishigaki and Okinawa individuals. This result is rather difficult to explain by one-directional settlement of individuals from Ishigaki to Okinawa. Rather, bidirectional exchange between the two islands may provide more a reasonable explanation for the results. If so, surface ocean currents in the Ryukyu Archipelago are more complex than previously assumed, including currents that allow larval movement from Okinawa to Ishigaki. A similar example has been shown in a coral species (Zayasu *et al.* 2016). Marine currents in the Ryukyu Archipelago should be explored in the future in relation to marine invertebrate larval dispersal.

#### Acanthaster planci:

**Figure 5:**
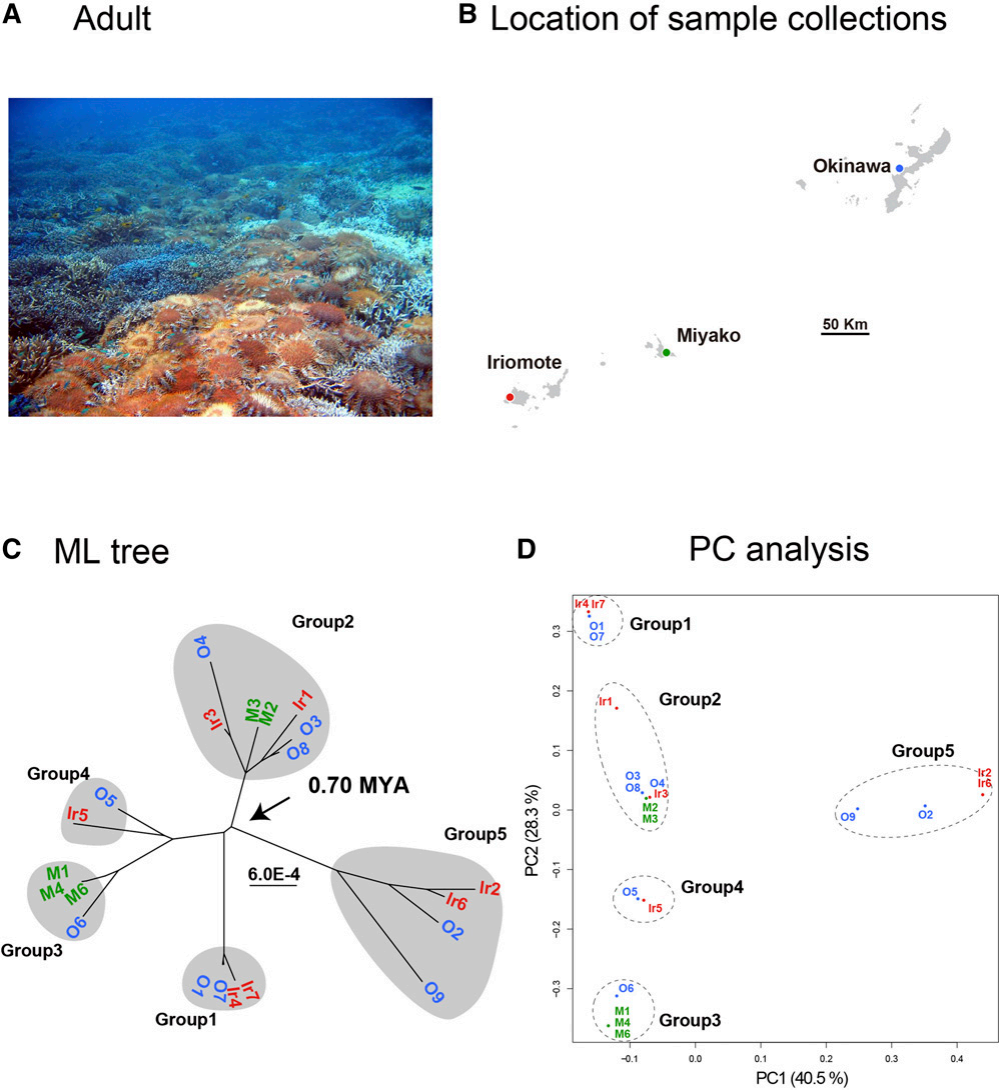
Population genetics of *Acanthaster planci*. (a) Many adults covering corals. (b) Location of collecting sites in the Ryukyu Archipelago, Japan. (c) Population genetics showing five different populations that diverged approximately 0.7 MYA. The five distinct population comprise mixtures of individuals from Okinawa, Miyako, and Iriomote Islands. (d) PCA of the populations, supporting the population genetic profile shown in (c).

We attempted to develop complete mitochondrial DNA sequences for 12, 6, and 8 specimens from Okinawa, Miyako, and Iriomote Islands, respectively, but we succeeded for only 9, 5, and 7 individuals (Figure 5a, b). Therefore, we performed population genomics analyses on 21 specimens using the alignment including 268 variable sites of 16,230-16,282-bp sequences. This resulted in a profile quite different from those of *As. amurensis* and *L. laevigata* (Figure 5c). First, the divergence date of *A. planci* was estimated to be 0.7 MYA (Figure 2), a much more recent event compared to the separation of *As. amurensis* and *L. laevigata*. In addition, the analysis suggested the divergence of five populations, each comprising a small number (2∼6) of individuals (Figure 5c). Branch lengths of the five groups from ancestral divergence points were also very short. PCA also supported the existence of five small populations (Figure 5d). This suggests a genetic bottleneck in the history of *A. planci* in the Ryukyu Archipelago, which may reflect periodic COTS outbreaks in this area.

As in the case of *L. laevigata*, each of the five groups comprised individuals from all three islands (Figure 5c, d). For example, G2 was composed of three individuals from Okinawa (O3, 4 and 8), two from Miyako (M2 and 3), and two from Iriomote (Ir1 and 3). Even though Okinawa Island is separated from Iriomote Island by more than 450 km, this indicates significant gene flow among the three Islands, *i.e.*, frequent larval dispersal from all three islands. If this is correct, an outbreak at Ishigaki and Iriomote could trigger a similar event in Okinawa within several years, or *vice versa*. Such frequent larval exchange might partially explain periodic local outbreaks in the Ryukyu Archipelago since the 1950s.

Previous studies have demonstrated discrete populations of COTS in various locations in the Indo-Pacific Ocean (Nishida and Lucas 1988; Yasuda *et al.* 2009; Timmers *et al.* 2012; Vogler *et al.* 2013; Yasuda *et al.* 2015; Tusso *et al.* 2016). The present study offers insights into COTS population genetics in the Ryukyu Archipelago. That is, COTS have likely experienced recent genetic bottlenecks that may be associated with their periodic outbreaks. Ocean currents in the Ryukyu Archipelago appear more complicated than previously assumed, consisting not just of the southwest-to-northeast Kuroshio Current, but comprising multi-directional currents, including flows in the opposite direction. This might play a role in multidirectional gene flow in this area. Influence of these ocean currents is not investigated for marine organisms except for a few studies (Ogoh and Ohmiya 2005; Shinzato *et al.* 2015; Soliman *et al.* 2016; Zayasu *et al.* 2016). Moreover, population genetic structures may reflect differences in the duration of the pelagic larval stage as shown in Yorifuji *et al.* (2012). Thus, further studies will be required to better understand ecological and reproductive mechanisms of COTS outbreaks in this area.
